# Time to initiation of breastfeeding and neonatal mortality and morbidity: a systematic review

**DOI:** 10.1186/1471-2458-13-S3-S19

**Published:** 2013-09-17

**Authors:** Amanda K Debes, Anjalee Kohli, Neff Walker, Karen Edmond, Luke C Mullany

**Affiliations:** 1 Department of International Health, Johns Hopkins Bloomberg School of Public Health, Baltimore, MD, USA; 2 School of Pediatrics and Child Health, University of Western Australia, Crawley, WA, Australia

## Abstract

**Background:**

Early breastfeeding is defined as the initiation of breastfeeding within twenty four hours of birth. While the benefits of breastfeeding have been known for decades, only recently has the role of time to initiation of breastfeeding in neonatal mortality and morbidity been assessed.

**Objective:**

To review the evidence for early breastfeeding initiation practices and to estimate the association between timing and neonatal outcomes.

**Methods:**

We systematically reviewed multiple databases from 1963 to 2011. Standardized abstraction tables were used and quality was assessed for each study utilizing the Grading of Recommendations Assessment, Development and Evaluation (GRADE) methodology. Three meta-analyses were conducted for mortality among babies surviving to 48 hours.

**Results:**

We identified 18 studies reporting a direct association between early breastfeeding initiation and neonatal mortality and morbidity outcomes. The results of random effects analyses of data from 3 studies (from 5 publications) demonstrated lower risks of all-cause neonatal mortality among all live births (RR = 0.56 [95% CI: 0.40 – 0.79]) and among low birth weight babies (RR=0.58 [95% CI: 0.43 – 0.78]), and infection-related neonatal mortality (RR = 0.55 [95% CI: 0.36 – 0.84]). Among exclusively breastfed infants, all-cause mortality risk did not differ between early and late initiators (RR = 0.69 [95% CI: 0.27 – 1.75]).

**Conclusions:**

This review demonstrates that early breastfeeding initiation is a simple intervention that has the potential to significantly improve neonatal outcomes and should be universally recommended. Significant gaps in knowledge are highlighted, revealing a need to prioritize additional high quality studies that further clarify the specific cause of death, as well as providing improved understanding of the independent or combined effects of early initiation and breastfeeding patterns.

## Background

Millennium Development Goal (MDG) 4 aims to reduce under-five mortality by two-thirds, globally by 2015. Since the goals were set the under-five mortality rate has dropped by 35 percent, and the rate of decline in under-five mortality continues to improve, from 1.9% per year from 1990 – 2000 to 2.6% from 2000 – 2009 [1]. However, the global community is still behind schedule to meet the 2015 deadline. The percent of under-five deaths that occur during the neonatal period, the first month of life, has increased from 10% in 1990 to 40% as of 2010 [2]; deaths in this period are primarily due to preterm birth, intrapartum-related hypoxic events, and infections [2]. Thus, the work to reduce under-five mortality is increasingly focused on neonatal mortality in order to achieve the overall reductions necessary to meet MDG 4.

Studies on the benefits of breastfeeding have demonstrated substantial benefits for child health [3,4]. The recommendation for exclusive breast-feeding in newborns and infants has a long history, and research has demonstrated that breastfeeding protects against many illnesses and infectious diseases, including reducing the risk of diarrhea [5], respiratory infections especially pneumonia [6], meningitis [6], and neonatal sepsis [6-9]. Attention has largely focused on the protective effects of breastfeeding in the first year of life, and in particular, greater protection appears to be conferred in the first six months of life [10]. Only recently, however, has attention been directed towards both the pattern of breastfeeding as well as the timing of initiation of breastfeeding and the effects on neonatal morbidity and mortality [11]. While research assessing the importance of breastfeeding over the past century has reinforced the protective effect of breastfeeding, including in the neonatal period, few studies have assessed the impact of the ***time to breast feeding initiation*** on infant and neonatal mortality and morbidity. We conducted a systematic review to estimate the relationship between early initiation of breastfeeding (<24 hours after birth) on neonatal (<28 days) mortality and morbidity.

## Methods

**Figure 1:**
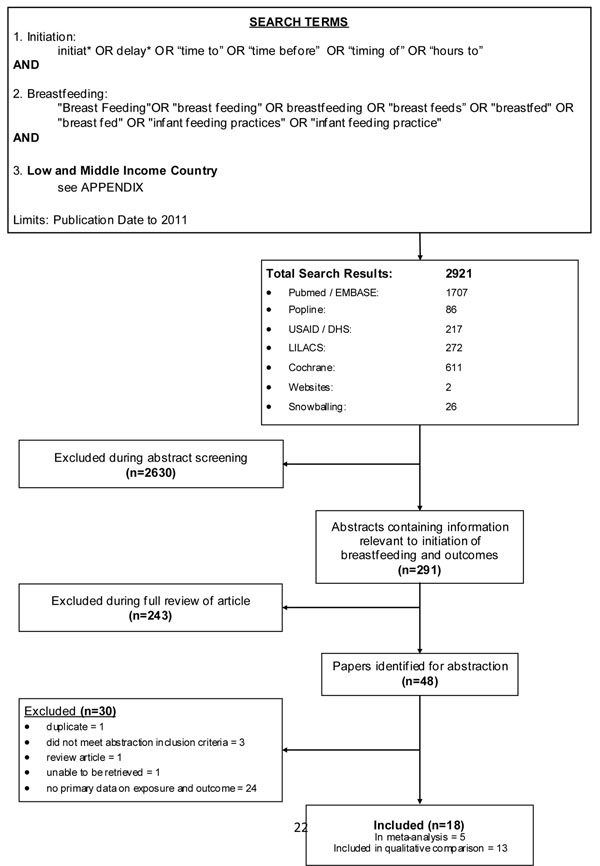
Flow diagram of systematic review

A systematic review was performed on all literature published from 1963 to 2011 to identify studies evaluating the early initiation of breastfeeding and its association with neonatal outcomes. Pubmed, EMBASE, Popline, USAID reports, LILACS database and Cochrane Libraries were searched and publications in any language were included. We conducted our initial search of Pubmed, Embase, Popline and USAID reports on June 5, 2011, and two updated searches on November 18, 2011 of the LILACS database and on December 9, 2011 (the Cochrane Libraries). Additionally, several key websites were reviewed to identify workshops or reports relating to breastfeeding initiation. Combinations of the following search terms were used in these searches: “breastfeeding”, “initiation”, “timing”, “delay”, “neonatal” and “infant.” The terms specifically targeting “morbidity” or “mortality” were not included to allow for a broader search; this method was utilized to reduce any unintentional filtering of studies that might have reported an unexpected outcome or phrased the results in a unique manner. These searches were initially performed to review literature from all countries to ensure publications from all settings were included. Subsequently, the search was conducted with a low- and middle-income country filter to further focus the search (see Figure 1 for search terms and Additional File 1 for low and middle income countries filter terms). Finally, we reviewed the references of all relevant papers to ensure that all pertinent papers were identified.

Articles were initially screened based on title and abstract, selecting for studies specific to time to breastfeeding and infant (<1 year of age) or neonatal (<28 days) outcomes of morbidity and mortality. Subsequently, publications selected for full review were evaluated using additional inclusion and exclusion criteria. Only articles that directly linked primary data on exposure (time to initiation of breastfeeding) to one or more infant/neonatal outcomes (mortality or morbidity) were retained. Morbidity outcomes included infectious diseases, diarrhea, sepsis, malnutrition, omphalitis, weight loss, and growth. Many studies reported breastfeeding initiation time as one of the variables accounted for when assessing neonatal mortality and morbidities, but if no attempt was made to compare initiation time with the outcomes the study was excluded. We considered prospective studies, including randomized control trials, observational studies and cohort studies. Retrospective studies were considered but rated with a lower grade due to the biases such studies may impose on assessment of the relationship.

We excluded any studies that did not fulfill the inclusion criteria, as well as duplicate studies. We excluded studies with data presented in a form that was unclear or difficult to interpret [12,13]. Additionally, for studies in which one or more neonatal outcomes were compared across multiple exposures, we only abstracted data on the effects of time to initiation of breastfeeding and the reported morbidity. All aspects of screening, including abstracts review, full article review and abstraction of relevant studies were completed using double-data abstraction into a standardized form.

In this review, breastfeeding initiation time data was abstracted and, if possible, re-organized according to our target definition of early initiation of breastfeeding: i.e. within 24 hours of birth (“early”) or equal to or after24 hours since birth (“late”). For the purposes of reorganization, data reported in multiple shorter time intervals between birth and 24 hours were combined (i.e. <2 hours, 2-24 hours), and compared to late initiation. In some instances, data reported used an alternate binary cutoff (i.e. <2 hours vs. >2 hours, or <6 hours vs. >6 hours, etc.), and no re-organization of the data was possible. We did not stratify results according to shorter time periods to breast feeding initiation, such as a period of <12 hours or <1 hour as these data are not consistently reported in the literature.

All studies that met the inclusion criteria for the full article screen were abstracted using a standardized form. The data were compiled and ranked according to the outcome of measure using an assessment of outcome quality derived from the Child Health Epidemiology Reference Group (CHERG) guidelines. Per the CHERG guidelines, the quality of evidence provided in each study was scored as low, medium or high quality [14]; the definitions for each grade is shown in Table 1. We only included papers with a high ranking in any meta-analysis of the association between initiation of breastfeeding and neonatal outcomes. In addition to requiring that the study presented a quantified estimation of the relationship between breastfeeding initiation time and the outcome, the ranking system placed a high value on papers that 1) accounted for reverse causality, 2) adjusted for important confounders including gestational age and low birth weight, and 3) were prospective in design.

**Table 1:** Criteria used to rank included studies as high, medium, and low quality for inclusion in meta-analysis

Ranking	Quantified BF – outcome relationship	Accounting for Reverse Causality	Adjustment for potential confounders	Study Design
**HIGH**	Required. Must cover/quantify the outcome relationship during the neonatal period	Required. Must remove from analysis babies that might have not been breastfed early as a result of their status/illness	Required. Must adjust for gestational age and/or low birth weight. Adjustment for other confounders desirable	Prospective cohort, RCT
**MEDIUM**	Required. Must cover/quantify the outcome relationship during the neonatal period	No	Required. Must adjust for gestational age and/or low birth weight. Adjustment for other confounders desirable	Prospective cohort, RCT
**LOW**	Required. Must cover/quantify the outcome relationship during the neonatal period	No	No	Case control, retrospective

It is important to establish temporality prior to the onset of illness or death in order to properly measure the association between breastfeeding initiation time and this outcome. For examination of the association with mortality, only studies that excluded deaths occurring within the first 48 hours after birth were given high ranking. Additionally, studies were considered as either high or medium grade if the authors adjusted for low birth weight and prematurity. This is necessary to control for selection bias in women who either do not initiate breastfeeding, delay initiation of breastfeeding, or partially breastfeed as a direct result of the health status of the infant [10]. This type of selection bias is prone to increasing the perceived benefits of breastfeeding initiation time on child survival. Studies that examine timing of breastfeeding and infant health outcomes should adjust for reverse causality (i.e. baby’s or mother’s health status) to avoid over-reporting of the benefits of breastfeeding [15]. Studies should additionally adjust for infant health at birth or in the proceeding days to account for medications or liquids that might be given to treat illness. Further, the feeding practice at death or during illness may not be the feeding pattern practiced prior to this outcome [10]. To account for these types of biases, studies were weighted as low if the authors did not account for low-birth weight, prematurity or reverse causality (due to congenital abnormalities or any other serious illness that is not related to the outcomes of interest). Studies that were not prospective were ranked as low if they estimated the relationship, but were excluded if they lacked this information

The morbidity-focused data and/or presented analyses were of insufficient quality to achieve a HIGH quality rating, thus our meta- analysis does not include quantitative estimation of the possible protective benefits of early initiation of breastfeeding associated with the reduction in morbidities. Therefore, we present these results qualitatively with main conclusions and comments as supportive evidence for the overall benefit of early breastfeeding. Specific morbidities included in this qualitative presentation include neonatal hypothermia, malnutrition indicators (weight for age (WAZ), length-for- age (LAZ), and weight-for-length (WLZ)), neonatal weight loss, omphalitis, hypoglycemia, and diarrhea (persistent and acute diarrhea). Several studies were rated as MEDIUM quality according to the criteria; however they were not targeting the same morbidity and thus could not be combined for a meta-analysis assessing the association between early initiation of breastfeeding and specific morbidities

According to CHERG standards, we abstracted measures of effect as well as 95% confidence intervals from all studies with a high ranking. In studies where relative risk (RR) was not the reported measure, authors were contacted and the adjusted relative risk measures were recalculated for use in this meta-analysis. We conducted a random-effects meta-analysis for a number of mortality-based outcomes:

1. Deaths from all causes within 28 days of birth among all babies surviving to 48 hours; Analyses were conducted a) among all live births; b) among babies <2500 grams at first weight measurement; and c) among babies exclusively breastfed.

2. Deaths from specific causes within 28 days of birth among babies surviving to 48 hours. Causes included a) “infection” (a more general non-specific categorization term including sepsis, meningitis, pneumonia, tetanus, diarrhea, dysentery, or other infectious diseases), b) sepsis-specific, c) birth asphyxia, and d) complications of premature delivery. Not all specific causes were available from each study.

The overall estimate for each outcome was calculated using the standard DerSimonian and Laird method with inverse variance weights [16]. These analyses were conducted using the user-written *metan* suite of commands available in STATA version 11.0 (Stata Corp., College Station, TX).

## Results

Our search identified 2921 papers, with an additional 26 studies identified by snowball searching in which the relevant citations identified in full-review articles were retrieved and screened if applicable (Figure 1). After initial title and abstract screening, 291 articles were identified to have information relevant to time to initiation of breastfeeding and relevant outcomes. From the full article review of these 291 articles, 48 were evaluated to have low, medium or high quality data fitting the criteria for abstraction. Of these 48 articles, 30 were not suitable for data abstraction: 1 was a duplicate; 3 were erroneously approved for abstraction but did not fit criteria; 1 was not able to be retrieved for abstraction; 1 was a review of breast-feeding improvements to reduce neonatal mortality; 22 presented data on either time to initiation of breast feeding and neonatal morbidity or mortality, or both, but did not present primary data **and** a direct estimate of the association between our exposure of interest and a morbidity or mortality outcome; and 2 did not focus on neonatal outcomes when assessing time to breastfeeding initiation [17,18]. After exclusions, the final set of included publications totaled 18 (from 14 distinct studies)and included 11 prospective cohort analyses [11,19-28] (7 distinct studies), 3 unmatched case-control studies [29-31], 2 cross-sectional surveys [32,33], 1 matched case-control [34], and 1 randomized trial [35]. Data for these studies were collected in South Asia (6 studies, n=8 included publications), sub-Saharan Africa (6 studies, n=8 included publications), Northern Africa (n=1) and Europe (n=1).

### *1.* Breastfeeding initiation time and mortality outcomes

Three secondary analyses of data collected within the context of large cluster-randomized trials of maternal (vitamin A supplementation [36]) and neonatal interventions (vitamin A supplementation [37], chlorhexidine skin [38] and cord cleansing [39]) examined timing of breastfeeding initiation and mortality outcomes; each trial included >10,000 live births. Table 2 presents the study-specific estimates and 95% confidence intervals for the association between timing of breastfeeding and all-cause mortality among all infants and among the subgroup of infants that were low birth weight (<2500 g), while study-specific estimates for the association with specific causes of death are summarized in Table 3. A summary of the combined estimates is provided in Table 4. For each study and for each outcome, analyses were restricted to deaths occurring after 48 hours, and adjusted for low birth weight, gestational age, and other confounders (these varied by study).

**Table 2:** Summary of included studies presenting estimates of association between early breastfeeding initiation and all-cause mortality

Study, Location	Sample Size	Study	Def of BF Exposure	Comment/Conclusions	Grade
**All-cause neonatal mortality, among all infants surviving to 48 hours or more**					

Mullany [11], Nepal, community	22,838	prospective cohort	early vs. late	Early breastfeeding initiation was associated with a lower risk of mortality: RR=0.71 (0.54, 0.93)	HIGH
Edmond [20], Ghana, community	10,947	prospective cohort	early vs. late	Early breastfeeding initiation was associated with a lower risk of mortality: RR=0.43 (0.31, 0.61)	HIGH
Garcia [24], India, community	10,464	prospective cohort	early vs. late	Early breastfeeding initiation was associated with a lower risk of mortality: RR=0.56 (0.32, 0.97)	HIGH
Bamji [31], India, community	378	case control	early vs. late	Reported a significant association between early initiation and a reduction in neonatal mortality	LOW

**All-cause neonatal mortality, among low birth weight infants surviving to 48 hours or more**					

Mullany [11], Nepal, community	22,838	prospective cohort	early vs. late	Early breastfeeding initiation was associated with lower risk of mortality: RR=0.63 (0.44, 0.90)	HIGH
Edmond [22], Ghana, community	10,947	prospective cohort	early vs. late	Early breastfeeding initiation was associated with lower risk of mortality: RR=0.37 (0.14, 0.98)	HIGH
Garcia [24], India, community	10,464	prospective cohort	early vs. late	Mortality was lower among those breastfed early, but not statistically significant: RR=0.54 (0.28, 1.04)	HIGH

**All-cause neonatal mortality, among exclusively breastfed infants surviving to 48 hours or more**					

Mullany [11], Nepal, community	22,838	prospective cohort	early vs. late	No evidence that early breastfeeding was associated with mortality: RR=1.21 (0.49, 2.98)	HIGH
Edmond [22], Ghana, community	10,947	prospective cohort	early vs. late	Early breastfeeding initiation was associated with lower risk of mortality: RR=0.46 (0.29, 0.72)	HIGH

**Table 3:** Summary of included studies presenting estimates of association between breastfeeding initiation and cause-specific mortality

Study/References	Ghana	Nepal	India
**Author (Year)**	**Edmond (2006)**	**Mullany (2008)**	**Garcia (2011)**

**Location**	Community	Community	Community
**Sample Size**	10,947	22,838	10,464
**Design**	Prospective cohort	Prospective cohort	Prospective Cohort
**Definition of Exposure**	Early vs. late	Early vs. late	Early vs. late
**Cause of Death Outcome**			
- **Infection**	0.39 (0.26 – 0.61)	0.70 (0.46 – 1.06)	0.68 (0.30 – 1.54)
- **Sepsis**	0.38 (0.20 – 0.83)	0.61 (0.38 – 0.97)	0.20 (0.07 – 0.60)
- **Birth Asphyxia**	0.45 (0.15 – 1.41)	0.48 (0.12 – 1.98)	0.79 (0.11 – 5.94)
- **Premature**	0.73 (0.24 – 1.45)	0.44 (0.19 – 1.00)	n/a
**Grade**	HIGH	HIGH	HIGH

**Table 4:** Summary of the type and quality of evidence for mortality outcomes

No of Studies	Design	Mortality outcome*	Consistency	Generalizability (location)	Relative Risk (95% CI)	Grade	Comments
3	prospective cohort studies	All causes, among all live births	marginal heterogeneity from meta analysis (p=0.076); all studies show a benefit	South Asia, West Africa	0.56 (0.40 – 0.79)		Random effects meta analysis
3	prospective cohort studies	All causes, among low birth weight babies	Q test for heterogeneity from meta analysis (p=0.585); two of three studies show a benefit	South Asia, West Africa	0.58(0.43 – 0.78)		Random effects meta analysis
3	prospective cohort studies	Infection-related causes, among all live births	Q test for heterogeneity from meta analysis (p=0.134); one of three studies shows a benefit	South Asia, West Africa	0.55 (0.36 – 0.84)		Random effects meta analysis
3	prospective cohort studies	Sepsis-specific mortality, among all live births	Q test for heterogeneity from meta analysis (p=0.138); all studies show a benefit	South Asia, West Africa	0.42 (0.23 – 0.74)		Random effects meta analysis
3	prospective cohort studies	Birth asphyxia-specific mortality, among all live births	Q test for heterogeneity from meta analysis (p=0.887); zero of three studies show a benefit	South Asia, West Africa	0.50 (0.23 – 1.12)		Random effects meta analysis
2	prospective cohort studies	Prematurity-specific mortality, among all live births	Q test for heterogeneity from meta analysis (p=0.418); one of two studies show a benefit	South Asia, West Africa	0.56 (0.30, 1.02)		Random effects meta analysis

#### *1a.* Association with deaths from all causes

**Figure 2:**
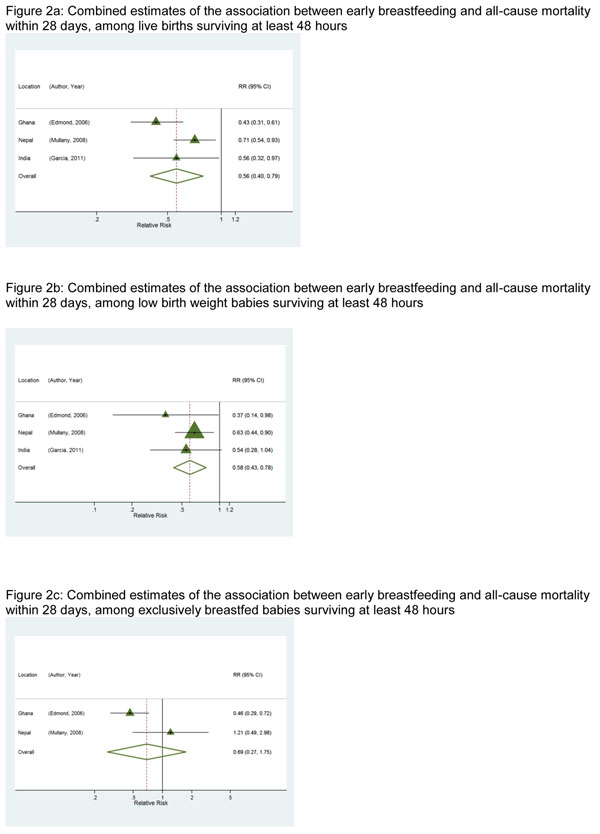
**a:** Combined estimates of the association between early breastfeeding and all-cause mortality within 28 days, among live births surviving at least 48 hours. **Figure 2b:** Combined estimates of the association between early breastfeeding and all-cause mortality within 28 days, among low birth weight babies surviving at least 48 hours. **Figure 2c:** Combined estimates of the association between early breastfeeding and all-cause mortality within 28 days, among exclusively breastfed babies surviving at least 48 hours

All three studies individually estimated a protective association between early initiation of breastfeeding and all-cause neonatal mortality among babies surviving the first 48 hours; the combined estimate of association indicated 44%(RR=0.56 [95% CI: 0.40 – 0.79]) lower risk of death. The overall estimate of association between breastfeeding initiation time and all-cause mortality among low birth weight babies demonstrated a 42% (RR=0.58 [95% CI: 0.43 – 0.78]) lower risk. When analysis of the association was restricted to babies that were exclusively breastfed (available for two studies only: Nepal, Ghana), study-specific estimates were substantially different, and overall there was no evidence of a protective benefit of early breastfeeding (RR=0.69 [95% CI: 0.27 – 1.75]). The study conducted in Ghana showed the greatest reduction in risk among all babies, among low birth weight babies, and among those exclusively breastfed. Forest plots for these analyses are found in Figures 2a, 2b, and 2c, respectively.

#### *1b.* Association with deaths from specific causes

**Figure 3:**
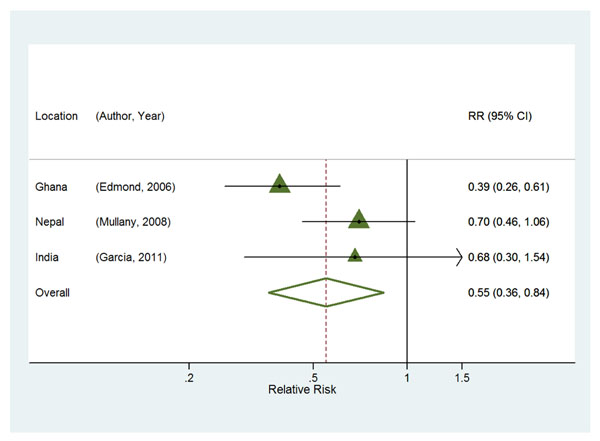
Combined estimates of the association between early breastfeeding and infection-related mortality outcomes within 28 days, among all live births surviving at least 48 hours

**Figure 4:**
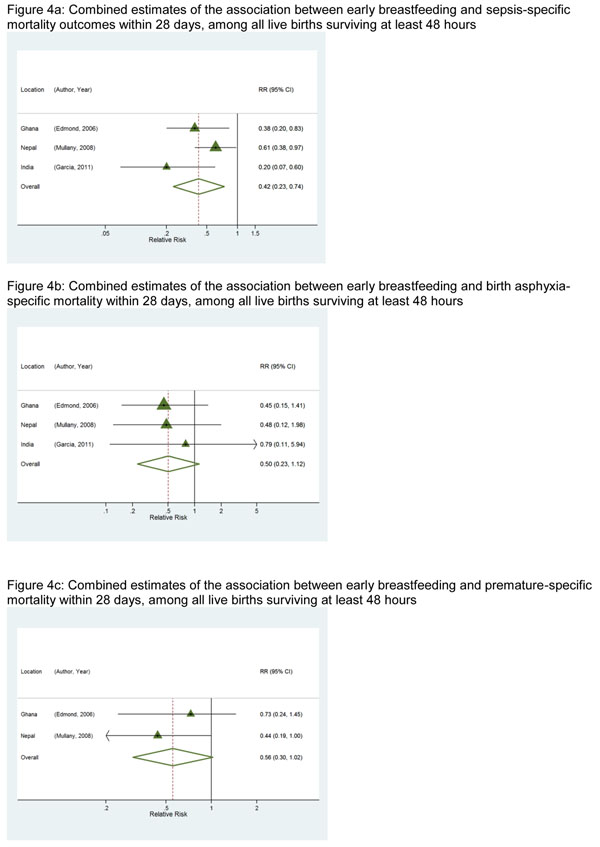
**a:** Combined estimates of the association between early breastfeeding and sepsis-specific mortality outcomes within 28 days, among all live births surviving at least 48 hours. **Figure 4b:** Combined estimates of the association between early breastfeeding and birth asphyxia-specific mortality within 28 days, among all live births surviving at least 48 hours. **Figure 4c:** Combined estimates of the association between early breastfeeding and premature-specific mortality within 28 days, among all live births surviving at least 48 hours

For the association between early breastfeeding initiation and infection-related mortality, the magnitude was similar to the all-cause mortality estimates (both overall and among low birth weight babies). The risk of death was 45% (RR=0.55 [95% CI: 0.36– 0.84]) lower among those breastfed early (Figure 3). When infection-related deaths were restricted to those classified in the individual studies as “sepsis” or “septicemia”, the risk of death was 58% (RR=0.42 [95% CI: 0.23 – 0.74]) lower (Figure 4a). Early initiation of breastfeeding was not associated with birth-asphyxia specific deaths (RR=0.50 [95% CI: 0.23 – 1.12], Figure 4b) or deaths due to complications of prematurity (RR=0.56 [95% CI: 0.30-1.02], Figure 4c).

#### *1c.* Breastfeeding initiation time and morbidity

Due, in part, to inconsistent definitions of early breastfeeding and varying presentation of data (e.g., reporting of time to initiation of breastfeeding not standardized, original data not available in publication), zero studies that examined the relationship between early breastfeeding and specific morbidities ranked as HIGH quality (Table 5). Nevertheless, several studies ranked as MEDIUM, demonstrating a protective effect of early breastfeeding on neonatal morbidities including reduced weight loss and hypothermia. For example, a matched case-control study in Turkey reported that infants with a weight loss of >10% (3.39 + 2.37h) initiated breastfeeding later than those who lost <10% (2.14 + 1.31h) after controlling for birth weight, gestational age and reverse causality [31]. This protective effect of early initiation of breastfeeding on reduced weight loss was also identified in a prospective case cohort study in Zaire (i.e. Democratic Republic of Congo) [23]. Yet, a number of small LOW quality studies found inconsistent associations between early breastfeeding and longer-term nutritional-indicators [29,32,33] A prospective cohort study in Nepal (ranked as MEDIUM) reported an association between early initiation of breastfeeding and lower incidence of neonatal hypothermia, after adjustment for confounders (prevalence rate ratio of 0.84 [95% CI: 0.77 – 0.93]) [27]. In a sub-analysis of the Nepal dataset, the authors did not detect an association between early breastfeeding and signs of omphalitis [25]. A smaller prospective study in Zanzibar supported the association between early breastfeeding (within 1 hour) and lower rates of moderate/severe omphalitis (RR=0.29 [95%CI: 0.11 – 0.74]) [26]. A small prospective cohort study of breastfeeding practices in Egypt reported lower rates of diarrhea among babies breastfed within 72 hours of birth [19]. While that study adjusted for reverse causality (i.e. infants with major congenital abnormalities and/or illnesses requiring hospitalization were excluded), low birth weight, gestational age and time intervals between birth and 72 hours after birth were not accounted for in the analysis. Finally, a study conducted in Malawi that randomly allocated the exposure of interest (i.e. breastfeeding initiation immediately after delivery) found that significantly fewer neonates that breastfed earlier had a temperature below <36.5 °C the day after delivery [35].

**Table 5:** Summary of included studies presenting estimates of association between early breastfeeding initiation and morbidity outcomes

Study, Location	Sample Size	Study Design	Def of BF Exposure	Comment/Conclusions	Grade
**Malnutrition WAZ**					

Appoh [29], Ghana, facility	110	case-control	early vs. late	Reported an association between early breastfeeding initiation and a reduction in underweight children	LOW
Kumar [33], India, community	217	cross sectional survey	<6h vs. >6h	Reported an association between early breastfeeding initiation and a reduction in underweight children	LOW

**Malnutrition WLZ**					

Engebretsen [32], Uganda, community	723	cross sectional survey	<2h vs 2-24h<2h vs>24h	There was no statistically significant relationship comparing >24hr&<24 hours to <2hours (reference)	LOW
Kumar [33], India, community	217	cross sectional survey	<6h vs. >6h	The infant feeding practices studied were not significantly associated with wasting	LOW

**Malnutrition, LAZ**					

Engebretsen [32], Uganda, community	723	cross sectional survey	<2h vs 2-24h<2h vs>24h	There was no statistically significant relationship comparing >24hr&<24 hours to <2hours (reference	LOW
Kumar [33], India, community	217	cross sectional survey	<6h vs. >6h	Reported a significant association between early breastfeeding initiation and reduction in stunting	LOW

**Early Weight Loss**					

Enzunga [23], Zaire, facility	330	prospective cohort	early vs. late	Reports a direct relationship between the delay in initiation of breast-feeding and subsequent weight loss	LOW
Caglar [34], Turkey, facility	90	prospective matched case control	mean time to bf initiation	Infants with a weight loss of >10% were significantly more likely to have received their first breastfeeding later than controls	MEDIUM

**Diarrhea**					

Badruddin [30], Pakistan, mixed facility/community	265	case-control	early vs. late	Reported a significant higher likelihood of late breastfeeding in cases (i.e. those with acute and/or persistent diarrhea)	LOW
Clemens [19], Egypt, community	198	prospective cohort	<72h vs. >72h	Reported a significant association between early breastfeeding initiation and reduction in diarrhea in the first six months of life	LOW

**Acute Diarrhea**					

Badruddin [30], Pakistan, mixed facility/community	265	case-control	early vs. late	Reported a significant higher likelihood of late breastfeeding in cases (i.e. those with acute diarrhea)	LOW

**Persistent Diarrhea**					

Badruddin [30], Pakistan, mixed facility/community	265	case-control	early vs. late	Reported a significant higher likelihood of late breastfeeding in cases (i.e. those with persistent diarrhea)	LOW

**Omphalitis**					

Mullany [26], Zanzibar, community	1653	prospective cohort	<1h vs. >=1h	Risk of omphalitis was 71% lower among babies breastfed within 1 hour	MEDIUM
Mullany [25], Nepal, community	17,198	prospective cohort	early vs. late	There was no statistically significant evidence to suggest that early breastfeeding initiation is protective against omphalitis	MEDIUM

**Hypoglycemia**					

Sasidharan [28], India, hospital	604	prospective cohort	early vs. late	Reported a significant association between early breastfeeding initiation and reduction in hypoglycemia	LOW

**Hypothermia**					

Mullany [27], Nepal, community	23,240	prospective cohort	early vs. late	The adjusted prevalence rate of hypothermia was 16% lower among babies for whom bf was initiated w/in 24hours	MEDIUM
Van den Bosch [35], Malawi, facility	160	randomized trial	Immediate vs. mother’s choice of initiation time	Reported a significant association between early initiation and a reduction in low body temperature	LOW

#### *1d.* Summary of the evidence for intervention effects

**Figure 5:**
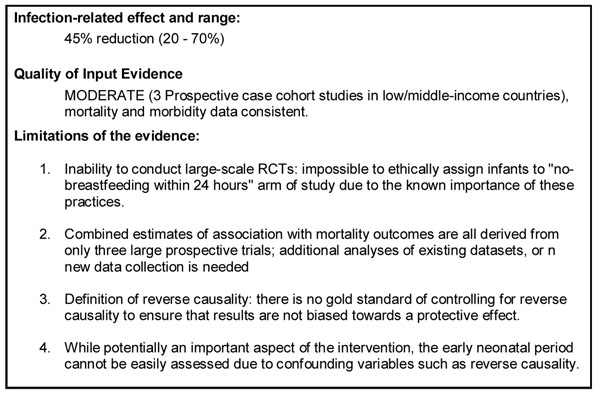
Infection-related mortality effect and quality grade of the estimate for the effect of early initiation of breastfeeding

To report the evidence of the neonatal mortality outcomes into an estimate of effectiveness of reducing cause-specific mortality, we applied the standard CHERG rules for generating estimated intervention effects for the use of the intervention in Lives Saved Tool (LiST). These rules are used as guidelines in the review to determine whether the evidence of effect resulting from the review justifies inclusion of the intervention in LiST. Rule 2 applies stating “if there is high- or moderate-quality evidence of effect on cause specific mortality…Then use the mortality effect,” [14]. The included studies provided strong evidence of association with statistical results from the three meta-analyses each contributing pooled estimates with p values of <0.001. However, due to the fact that these pooled estimates are based on observational studies rather than RCTs, the meta-analysis received a moderate quality of evidence score (See Figure 5).

## Discussion

Our data presented provides support for the protective effect of early breastfeeding initiation on death within the first 28 days, including all-cause mortality, deaths from infections, and deaths among low birth weight babies. We report a 44% (95% CI: 20 – 61%) lower risk of all-cause mortality within 28 days among live births surviving the first 48 hours of life, based on 3 prospective case cohort studies. Our findings are consistent with an additional small case control study with a long retrospective recall period, which estimated the odds of early breastfeeding among babies dying after day 1 were 87.0% lower than among survivors [31]. Deaths from all causes among low birth weight babies (42% lower [95% CI: 22 – 57%]) were also substantially lower among babies breastfed within 24 hours, but the magnitude and statistical strength did not differ from the overall result; this observation is true for both the combined estimate and for individual studies. Thus, while there is little evidence that the relative impact on mortality might differ between low birth weight and normal weight babies, the absolute benefit in terms of deaths averted through improved coverage of early breastfeeding would be greater among low birth weight babies, given higher underlying mortality risk.

The analyses of specific cause of death assist in our understanding of potential mechanisms by which early initiation may improve health outcomes among newborns. The association between breastfeeding initiation and the more general categorization of infection-related deaths was similar (RR=0.55) to that observed for all-cause mortality (RR=0.56); there are a number of explanations for this. One possible reason is that the verbal autopsy method used in these community trials does not always optimally classify cause of death, especially among newborns, as signs are often indistinguishable and/or overlapping among various causes. The more potential classifications that are grouped into a large categorization such as “infection-related deaths”, the greater the potential for the inclusion of babies with true causes that are not protected through early breastfeeding. The greater magnitude association between initiation time and the subset of infection related deaths that were sepsis-specific (RR=0.42) provides some support to this claim. Another possibility is that early breast-feeding initiation might additionally provide protection against some non-infectious causes of death, despite the current analyses not providing clear statistical evidence of such protection. Study-specific and combined estimates for birth asphyxia and deaths due to preterm complications (when available) were lower than 1.0; in the case of prematurity, the result was marginally statistically significant (p=0.06, RR=0.56 [95% CI: 0.30 – 1.02]). Furthermore, these analyses excluded deaths that occurred prior to 48 hours in an effort to minimize or eliminate reverse causality. Since the majority of deaths from these causes (preterm complications and birth asphyxia) occur within this immediate postpartum period, this effort to reduce reverse causality bias may also obscure some benefit in this group. In particular, the possibility of an association should not be disregarded for deaths due to preterm complications. Early breastfeeding is an integral part of kangaroo-mother-care, which has been shown to reduce mortality among hospitalized preterm babies [40].

The observed reduced mortality risk among babies breastfed early might be due to a number of factors. By displacing prelacteals, early initiation of breastfeeding can reduced risk of infections by decreasing the ingestion of infectious pathogens [19], and early milk is colostrum-rich, thus elevating exposure to immunoglobulins and lymphocytes that stimulate the humoral or cell-mediated immune system [41-43]. Through priming of the gastrointestinal tract, early breastfeeding can decrease intestinal permeability and the likelihood of translocation of infectious pathogens [44,45]. Further, skin-to-skin contact between the maternal-infant dyad may also stimulate the mucosa-associated lymphoid tissue system [46,47].

Our review of the literature includes a summary of analyses of neonatal or early infant morbidities. While these indicate some additional support for the potential benefit of early initiation, the studies are generally small in total size, do not adequately account for reverse causality, are inconsistent in the handling of important confounders such as gestational age or low birth weight, and, in some cases, are retrospective in design. The trend in Table 5 toward a protective association of early breastfeeding across multiple morbidities provides some support for the relationship, but the lack of high-quality analyses warrants caution and indicates a need for additional high-quality research in the area.

There are numerous limitations to the current review; these limitation results in a **moderate** rating of input evidence per CHERG guidelines (Box 1). First, we have only three main studies providing data for the analyses of mortality outcomes. There are some strengths to these studies, including multiple regions (South Asia and West Africa), prospective data collected in high-quality community-based randomized trials, and large sample size. However, despite consistent approaches to handling reverse causality (including removal of early deaths, and accounting for health status of baby), and adjustment for confounding, there remains the possibility of residual confounding and continuing reverse causality bias. A further limitation to interpretation arises from the weakness of the cause-of-death data. Individual studies used different verbal autopsy tools, definitions, and approach to classification (i.e. hierarchical vs. multiple causes, physician-adjudicated vs. computer-algorithm, etc.). The broadly defined categorization of “infection-related” deaths was associated with early breastfeeding, but may be subject to greater risk of misclassification of true cause. Within this group, neonatal sepsis was the predominant cause; when restricted to such deaths, the magnitude of association was increased, but statistical strength decreased, given smaller numbers. While both Africa and South Asia are represented, the current analyses are not sufficiently diverse, and further research from more countries is needed. The majority of the papers presented in this article were presented qualitatively due to the low quality rating as many of the studies did not account for major sources of bias that might incorrectly increase the perceived benefit of early breastfeeding practices on reduced infant and neonatal morbidity and mortality [15]. Further, many of the studies with low quality ratings published raw data or estimates in a form insufficient for meta-analysis, reducing further the ability to qualitatively or quantitatively compare studies assessing similar morbidity outcomes.

Finally, our review does not sufficiently cover other critical aspects of breastfeeding practices and potential impact on neonatal outcomes. These aspects include provision of colostrum, provision of prelacteals, and different patterns of breastfeeding ranging from mother’s breast milk only (“exclusive”), to the provision of prelacteals (including, among others, water, water-based fluids, or milk-based fluids) followed by exclusive breastfeeding (sometimes termed “predominant”), to other breastfeeding patterns including complementary feeding to varying extent (sometimes termed “partial”). Given the strong correlation between timing of breastfeeding and pattern of breastfeeding, treating these as distinct interventions with separate and independent impact likely oversimplifies this interaction. For example, in rural Nepal, the odds of establishing an exclusive breastfeeding pattern were 8.1 times higher among babies breastfed early than those initiating after 24 hours [11]. The association between early breastfeeding and all-cause mortality among babies surviving to 48 hours was no longer significant when restricted to those that were exclusively breastfed, but study-specific estimates were inconsistent, and data were only available from two studies. While it is not yet possible to conclude an independent benefit of early initiation of breastfeeding among exclusively breastfed infants, early initiation might substantially increase exclusive breastfeeding especially in settings where the most likely deviation from exclusivity occurs through the provision of prelacteals in the first hours after birth.

Thus, teasing out the complex interactions between timing of breastfeeding and these patterns is not straightforward, especially given the limited number of datasets available. The benefits of colostrum are well documented; is the apparent protective effect of early breastfeeding initiation conferred by the earlier and more frequent exposure to colostrum? If attributable to some extent, how much so? To what extent do the apparent survival and health advantages of early breastfeeding work through other mechanisms such as improved thermal status conferred through contact with mother, or improved nutritional or immunological status? What role does early breastfeeding play in establishing medium to long-term positive breastfeeding patterns, including exclusivity? To answer these and other questions, further efforts in this domain require a larger pool of high-quality data to better assess the independent or combined effects of various aspects of breastfeeding practice on neonatal outcomes. The LiST Tool currently includes “breastfeeding promotion” and current data are insufficient to include early breastfeeding as an independent, additional intervention.

Data from randomized controlled trials are not and will not be available, as it would not be ethical to randomize infants to a delayed initiation of breastfeeding. However, our understanding of the magnitude and extent of the protective effects of early initiation of breastfeeding would be greatly advanced through further analyses of existing datasets, and/or the inclusion of high quality, prospective measurement of timing of breastfeeding (and other feeding practices) and neonatal outcomes in current or planned large scale epidemiological studies. Such efforts should be carefully designed and conducted, and include accurate characterization of outcomes (including time and cause of death) and adequate measurement of potential confounders in order to mitigate methodological problems that otherwise substantially limit interpretation of the association between breastfeeding and neonatal health.

## Conclusions

This literature review and meta-analysis emphasizes the importance of early breastfeeding initiation for the reduction of risk of infant and neonatal morbidity and mortality. These findings support a recommendation of early initiation of breastfeeding as an intervention to reduce neonatal mortality and morbidity in low and middle income countries. Priority research gaps include the need for additional high quality studies on the association with mortality risk, with further clarity of the specific causes, as well as improved quality studies assessing the protective effects against morbidities. We also need a better understanding of the relationship between early breast feeding initiation and establishment and maintenance of good breastfeeding patterns. We encourage continued research to further strengthen the recommendation for promotion of this intervention and to increase the accuracy of the estimate of impact.

## List of abbreviations used

GRADE: Grading of Recommendations Assessment, Development and Evaluation; CHERG: Child Health Epidemiology Reference Group; LiST: Lives Saved Tool; RR: Relative Risk; CI: confidence interval.

## Competing interests

There authors declare that they have no competing interests.

## Authors’ contributions

AKD contributed to the data collection, systematic review, meta-analysis and wrote the manuscript. AK contributed to the systematic review and provided a critical review of the manuscript. NW contributed to the overall concept of the study and provided oversight of data collection. KE provided inputs on the overall concept, interpretation of the results and a review of the manuscript. LCM provided guidance on the inputs and overall concept, oversight of data collection, interpretation of the results and a critical review of the manuscript. All authors read and approved the final manuscript.

## Supplementary Material

Additional file 1 Search terms used in analyses. Click here for file

## Declaration

The publication costs for this supplement were funded by a grant from the Bill & Melinda Gates Foundation to the US Fund for UNICEF (grant 43386 to "Promote evidence-based decision making in designing maternal, neonatal, and child health interventions in low- and middle-income countries”). The Supplement Editor is the principal investigator and lead in the development of the Lives Saved Tool (LiST), supported by grant 43386. He declares that he has no competing interests.

This article has been published as part of *BMC Public Health* Volume 13 Supplement 3, 2013: The Lives Saved Tool in 2013: new capabilities and applications. The full contents of the supplement are available online at http://www.biomedcentral.com/bmcpublichealth/supplements/13/S3.
