# Supplementary material for: Time to initiation of breastfeeding and neonatal mortality and morbidity: a systematic review
Source: BMC Public Health. 2013 Sep 17;13(Suppl 3):S19. doi: 10.1186/1471-2458-13-S3-S19 (PMC3847227; doi:10.1186/1471-2458-13-S3-S19)
Supplement: Additional file 1 — Search terms used in analyses. [file 1471-2458-13-S3-S19-S1.docx]

**PubMed, Low and Middle Income countries**

Albania[tiab] OR Algeria[tiab] OR "American Samoa"[tiab] OR "Antigua and Barbuda"[tiab] OR Argentina[tiab] OR Azerbaijan[tiab] OR Belarus[tiab] OR "Bosnia and Herzegovina"[tiab] OR "Bosnia-Herzegovina"[tiab] OR Botswana[tiab] OR Brazil[tiab] OR Bulgaria[tiab] OR Chile[tiab] OR Colombia[tiab] OR "Costa Rica"[tiab] OR Cuba[tiab] OR Dominica[tiab] OR Dominican Republic[tiab] OR Fiji[tiab] OR Gabon[tiab] OR Grenada[tiab] OR Iran[tiab] OR Jamaica[tiab] OR Kazakhstan[tiab] OR Lebanon[tiab] OR Libya[tiab] OR Lithuania[tiab] OR Macedonia[tiab] OR Malaysia[tiab] OR Mauritius[tiab] OR Mayotte[tiab] OR Mexico[tiab] OR Montenegro[tiab] OR Namibia[tiab] OR Palau[tiab] OR Panama[tiab] OR Peru[tiab] OR Romania[tiab] OR Russia[tiab] OR "Russian Federation"[tiab] OR Serbia[tiab] OR Seychelles[tiab] OR "South Africa"[tiab] OR "Saint Kitts and Nevis"[tiab] OR "St Kitts and Nevis"[tiab] OR "Saint Vincent and the Grenadines"[tiab] OR "St Vincent and the Grenadines"[tiab] OR Suriname[tiab] OR Turkey[tiab] OR Uruguay[tiab] OR Venezuela[tiab] OR Angola[tiab] OR Armenia[tiab] OR Belize[tiab] OR Bhutan[tiab] OR Bolivia[tiab] OR Cameroon[tiab] OR "Cape Verde"[tiab] OR China[tiab] OR Congo[tiab] OR "Cote d'Ivoire"[tiab] OR "Ivory Coast"[tiab] OR Djibouti[tiab] OR Ecuador[tiab] OR Egypt[tiab] OR "El Salvador"[tiab] OR Georgia[tiab] OR Guatemala[tiab] OR Honduras[tiab] OR Indonesia[tiab] OR India[tiab] OR Iraq[tiab] OR Jordan[tiab] OR Kiribati[tiab] OR Kosovo[tiab] OR Lesotho[tiab] OR Maldives[tiab] OR "Marshall Islands"[tiab] OR Micronesia[tiab] OR Moldova[tiab] OR Mongolia[tiab] OR Morocco[tiab] OR Nicaragua[tiab] OR Nigeria[tiab] OR Pakistan[tiab] OR "Papua New Guinea"[tiab] OR Paraguay[tiab] OR Philippines[tiab] OR Samoa[tiab] OR "Sao Tome and Principe"[tiab] OR Senegal[tiab] OR "Sri Lanka"[tiab] OR Sudan[tiab] OR Swaziland[tiab] OR "Syrian Arab Republic"[tiab] OR Syria[tiab] OR Thailand[tiab] OR "Timor-Leste"[tiab] OR "East Timor"[tiab] OR Tonga[tiab] OR Tunisia[tiab] OR Turkmenistan[tiab] OR Tuvalu[tiab] OR Ukraine[tiab] OR Uzbekistan[tiab] OR Vanuatu[tiab] OR Vietnam[tiab] OR "West Bank and Gaza"[tiab] OR Yemen[tiab] OR Afghanistan[tiab] OR Bangladesh[tiab] OR Benin[tiab] OR "Burkina Faso"[tiab] OR Burundi[tiab] OR Cambodia[tiab] OR "Central African Republic"[tiab] OR Chad[tiab] OR Comoros[tiab] OR "Democratic Republic of the Congo"[tiab] OR "Congo"[tiab] OR Eritrea[tiab] OR Ethiopia[tiab] OR Gambia[tiab] OR Ghana[tiab] OR Guinea[tiab] OR "Guinea-Bissau"[tiab] OR Haiti[tiab] OR Kenya[tiab] OR Korea[tiab] OR "Kyrgyz Republic"[tiab] OR "Kyrgyzstan"[tiab] OR "Lao PDR"[tiab] OR "Laos"[tiab] OR Liberia[tiab] OR Madagascar[tiab] OR Malawi[tiab] OR Mali[tiab] OR Mauritania[tiab] OR Mozambique[tiab] OR Myanmar[tiab] OR Nepal[tiab] OR Niger[tiab] OR Rwanda[tiab] OR "Sierra Leone"[tiab] OR "Solomon Islands"[tiab] OR Somalia[tiab] OR Tajikistan[tiab] OR Tanzania[tiab] OR Togo[tiab] OR Uganda[tiab] OR Zambia[tiab] OR Zimbabwe[tiab] OR melanesia[tiab] OR "albania"[MeSH Terms] OR "algeria"[MeSH Terms] OR "American Samoa"[mh] OR "Antigua and Barbuda"[mh] OR "argentina"[MeSH Terms] OR "azerbaijan"[MeSH Terms] OR "republic of belarus"[MeSH Terms] OR "Bosnia-Herzegovina"[mh] OR "botswana"[MeSH Terms] OR "brazil"[MeSH Terms] OR "bulgaria"[MeSH Terms] OR "chile"[MeSH Terms] OR "colombia"[MeSH Terms] OR "Costa Rica"[mh] OR "cuba"[MeSH Terms] OR "dominica"[MeSH Terms] OR "dominican republic"[MeSH Terms] OR "fiji"[MeSH Terms] OR "gabon"[MeSH Terms] OR "grenada"[MeSH Terms] OR "iran"[MeSH Terms] OR "jamaica"[MeSH Terms] OR "kazakhstan"[MeSH Terms] OR "lebanon"[MeSH Terms] OR "libya"[MeSH Terms] OR "lithuania"[MeSH Terms] OR "macedonia (republic)"[MeSH Terms] OR "malaysia"[MeSH Terms] OR "mauritius"[MeSH Terms] OR "comoros"[MeSH Terms] OR "mexico"[MeSH Terms] OR "montenegro"[MeSH Terms] OR "namibia"[MeSH Terms] OR "palau"[MeSH Terms] OR "panama"[MeSH Terms] OR "peru"[MeSH Terms] OR "romania"[MeSH Terms] OR "russia"[MeSH Terms] OR "serbia"[MeSH Terms] OR "seychelles"[MeSH Terms] OR "South Africa"[mh] OR "Saint Vincent and the Grenadines"[mh] OR "suriname"[MeSH Terms] OR "turkey"[MeSH Terms] OR "uruguay"[MeSH Terms] OR "venezuela"[MeSH Terms] OR "angola"[MeSH Terms] OR "armenia"[MeSH Terms] OR "belize"[MeSH Terms] OR "bhutan"[MeSH Terms] OR "bolivia"[MeSH Terms] OR "cameroon"[MeSH Terms] OR "Cape Verde"[mh] OR "china"[MeSH Terms] OR "congo"[MeSH Terms] OR "Cote d'Ivoire"[mh] OR "djibouti"[MeSH Terms] OR "ecuador"[MeSH Terms] OR "egypt"[MeSH Terms] OR "El Salvador"[mh] OR ("georgia"[MeSH Terms] OR "georgia (republic)"[MeSH Terms]) OR "guatemala"[MeSH Terms] OR "honduras"[MeSH Terms] OR "indonesia"[MeSH Terms] OR "india"[MeSH Terms] OR "iraq"[MeSH Terms] OR "jordan"[MeSH Terms] OR "micronesia"[MeSH Terms] OR "yugoslavia"[MeSH Terms] OR "lesotho"[MeSH Terms] OR "indian ocean islands"[MeSH Terms] OR "micronesia"[MeSH Terms] OR "moldova"[MeSH Terms] OR "mongolia"[MeSH Terms] OR "morocco"[MeSH Terms] OR "nicaragua"[MeSH Terms] OR "nigeria"[MeSH Terms] OR "pakistan"[MeSH Terms] OR "Papua New Guinea"[mh] OR "paraguay"[MeSH Terms] OR "philippines"[MeSH Terms] OR "samoa"[MeSH Terms] OR "senegal"[MeSH Terms] OR "Sri Lanka"[mh] OR "sudan"[MeSH Terms] OR "swaziland"[MeSH Terms] OR "syria"[MeSH Terms] OR "syria"[MeSH Terms] OR "thailand"[MeSH Terms] OR "East Timor"[mh] OR "tonga"[MeSH Terms] OR "tunisia"[MeSH Terms] OR "turkmenistan"[MeSH Terms] OR "micronesia"[MeSH Terms] OR "ukraine"[MeSH Terms] OR "uzbekistan"[MeSH Terms] OR "vanuatu"[MeSH Terms] OR "vietnam"[MeSH Terms] OR "yemen"[MeSH Terms] OR "afghanistan"[MeSH Terms] OR "bangladesh"[MeSH Terms] OR "benin"[MeSH Terms] OR "Burkina Faso"[mh] OR "burundi"[MeSH Terms] OR "cambodia"[MeSH Terms] OR "Central African Republic"[mh] OR "chad"[MeSH Terms] OR "comoros"[MeSH Terms] OR "Democratic Republic of the Congo"[mh] OR "Congo"[mh] OR "eritrea"[MeSH Terms] OR "ethiopia"[MeSH Terms] OR "gambia"[MeSH Terms] OR "ghana"[MeSH Terms] OR "guinea"[MeSH Terms] OR "Guinea-Bissau"[mh] OR "haiti"[MeSH Terms] OR "kenya"[MeSH Terms] OR "korea"[MeSH Terms] OR "Kyrgyzstan"[mh] OR "Laos"[mh] OR "liberia"[MeSH Terms] OR "madagascar"[MeSH Terms] OR "malawi"[MeSH Terms] OR "mali"[MeSH Terms] OR "mauritania"[MeSH Terms] OR "mozambique"[MeSH Terms] OR "myanmar"[MeSH Terms] OR "nepal"[MeSH Terms] OR "niger"[MeSH Terms] OR "rwanda"[MeSH Terms] OR "Sierra Leone"[mh] OR "somalia"[MeSH Terms] OR "tajikistan"[MeSH Terms] OR "tanzania"[MeSH Terms] OR "togo"[MeSH Terms] OR "uganda"[MeSH Terms] OR "zambia"[MeSH Terms] OR "zimbabwe"[MeSH Terms] OR "melanesia"[MeSH Terms] OR "Africa"[Mesh:noexp] OR "Central America"[Mesh:noexp] OR "South America"[Mesh:noexp] OR "Latin America"[Mesh:noexp] OR "Asia"[Mesh:noexp] OR "Commonwealth of Independent States"[Mesh:noexp] OR "Pacific Islands"[Mesh:noexp] OR "Indian Ocean Islands"[Mesh:noexp] OR "Europe, Eastern"[Mesh:noexp] OR "Caribbean Region"[Mesh:noexp] OR "Atlantic Islands"[Mesh:noexp] OR "Middle East"[Mesh:noexp] OR "Developing countries"[mh] OR "developing countries"[tiab] OR "developing country"[tiab]
